# Supplementary material for: Who does tracing work for? Characteristics of clients successfully re-engaged in ART care in sub-Saharan Africa after a tracing intervention: A systematic review
Source: PLOS Glob Public Health. 2026 Mar 4;6(3):e0006026. doi: 10.1371/journal.pgph.0006026 (PMC12959690; doi:10.1371/journal.pgph.0006026)
Supplement: Attachment — Submitted filename: Rebuttal letter to Editor.docx [file pgph.0006026.s008.docx]

**Rebuttal letter to Editor’s concern about PGPH-D-25-02765**

September 29, 2025

To the Editor:

Our systematic review manuscript was rejected due to the restriction to English language sources. The Editor’s response states, “If you are able to revise the manuscript to respond to the concerns noted above, please refer to the original submission in the covering letter and accompany the submission by a rebuttal letter that responds to the comments made on the original manuscript.”

We would like to appeal this decision for the following reasons.

1. The search was not, in fact, limited to publications in English. It was our mistake to include this as a limitation in the Discussion section of the manuscript and in the systematic review protocol, as we did in fact include all languages in the search. We do apologize for this error. You will see that there is no language restriction in the search syntax (Supplementary file 2), nor was it mentioned in the Methods section of the paper. In fact, in our search in PubMed, we identified 179 foreign language source documents in the original search:

137 French

13 Spanish

9 German

6 Chinese

3 Turkish

3 Italian

2 Hungarian

2 Dutch

1 Norwegian

1 Croatian

1 Polish

1 Japanese

The search engine translated titles and abstracts for all of these sources into English, and they were reviewed during the abstract screening process. They were excluded for the following reasons:

6 wrong time frame/wrong study duration

5 wrong publication type

4 wrong study design

32 wrong population

132 wrong outcome

No sources were excluded due to a language restriction.

2. We note that PLOS Global Public Health has published multiple systematic reviews that were limited to English in 2025. These include, for example:

<https://doi.org/10.1371/journal.pgph.0004556>

<https://doi.org/10.1371/journal.pgph.0003336>

and many others.

3. Neither PLOS Global Public Health publication guidelines for systematic reviews nor the PRISMA guidelines appear to indicate that a language restriction is unacceptable.

For these reasons, we would like to ask the Editor to reconsider your decision not to review our manuscript.

With thanks,

Sydney Rosen
