# Supplementary material for: Who does tracing work for? Characteristics of clients successfully re-engaged in ART care in sub-Saharan Africa after a tracing intervention: A systematic review
Source: PLOS Glob Public Health. 2026 Mar 4;6(3):e0006026. doi: 10.1371/journal.pgph.0006026 (PMC12959690; doi:10.1371/journal.pgph.0006026)
Supplement: Attachment — Submitted filename: Response to Reviewers 2026 01 12.docx [file pgph.0006026.s009.docx]

**Response to reviewers of PGPH-D-25-02765, “Who does tracing work for? Characteristics of clients successfully re-engaged in ART care in sub-Saharan Africa after a tracing intervention: a systematic review”**

| **Comment** | **Response** |
| --- | --- |
| Editor’s comments |  |
| 1. We have noticed that you have cited Supplementary Table 3 in your manuscript. However, there are no corresponding files uploaded to the submission. Please upload them as separate files with the item type 'Supporting Information'. | We apologize for this oversight Supplementary Table 3 referred to our quality assessment of the included articles, which we had labeled as ‘Supplementary File 3’. We have corrected the reference in our revised submission. To accommodate some new supporting materials added for this revision, it is now labeled as Supplementary File 5. |
| 2. a. As required by our policy on Data Availability, please ensure your manuscript or supplementary information includes the following:   - A numbered table of all studies identified in the literature search, including those that were excluded from the analyses. - For every excluded study, the table should list the reason(s) for exclusion. - If any of the included studies are unpublished, include a link (URL) to the primary source or detailed information about how the content can be accessed.   b. A table of all data extracted from the primary research sources for the systematic review and/or meta-analysis. The table must include the following information for each study:   - Name of data extractors and date of data extraction - Confirmation that the study was eligible to be included in the review. - All data extracted from each study for the reported systematic review and/or meta-analysis that would be needed to replicate your analyses. - If data or supporting information were obtained from another source (e.g. correspondence with the author of the original research article), please provide the source of data and dates on which the data/information were obtained by your research group.   c. If applicable for your analysis, a table showing the completed risk of bias and quality/certainty assessments for each study or outcome.  Please ensure this is provided for each domain or parameter assessed. For example, if you used the Cochrane risk-of-bias tool for randomized trials, provide answers to each of the signalling questions for each study. If you used GRADE to assess certainty of evidence, provide judgements about each of the quality of evidence factor. This should be provided for each outcome.  d. An explanation of how missing data were handled. | Thank you. We have addressed these stipulations in the following ways:   a. We have included a numbered table with all studies identified in the search and reason(s) for exclusion (Supplementary File 4). Several of our studies had multiple reasons for exclusion. In reviewing this, we identified inconsistencies in the ways we prioritized exclusion criteria for those with multiple reasons. We harmonized these and have updated our PRISMA diagram (Figure 2 in the manuscript) accordingly.  b. We have included the table with all data extracted from the sources including names of data extractors and duration of data extraction as Supplementary File 3. During revision, we identified a missing row in Table 3; the corresponding data were already described in the manuscript, and the table has been updated for completeness.  c. We have attached our study quality assessment using the Joanna Briggs Institute critical appraisal toolkit as Supplementary File 5.  d. We have clarified how missing or unreported information was handled in the review. Characteristics or outcomes not reported in the original studies were recorded as “not reported (NR)”. As this systematic review was intended to describe reported characteristics, we did not conduct a meta-analysis, nor make assertions about missing data. This is now stated in the Methods (lines 211-212). The complete data extraction table for included studies, including fields recorded as NR, is provided as Supplementary File 3. |
| 3. We note that your Data Availability Statement is currently as follows: “All data relevant to the study are included in the article or uploaded as online supplemental information.”   Please confirm at this time whether or not your submission contains all raw data required to replicate the results of your study. Authors must share the “minimal data set” for their submission. PLOS defines the minimal data set to consist of the data required to replicate all study findings reported in the article, as well as related metadata and methods (<https://journals.plos.org/plosone/s/data-availability#loc-minimal-data-set-definition>). | All data reported are publicly available and we have indicated the fields that we have extracted in the points above. We note that this manuscript is a systematic review and that all input data are drawn from previously published sources. We have no additional data to share related to our manuscript. |
| **Reviewer’s comments** |  |
| This is a well-written manuscript with sound methodology and clear presentation. I recommend two minor revisions to strengthen the work: | Thank you for your thoughtful review of our work. |
| • Enhance the abstract by including specific patient characteristics associated with return to care (e.g., age ranges and key demographic details from your findings) to provide readers with immediate insight into which populations are most affected. | Thank you for this suggestion. We hesitate to include more specific quantitative information in the abstract due to the stated data limitations in the manuscript. The abstract does including the finding that older adults and females are more likely to return and notes associations with other characteristics. We believe that the abstract accurately summarizes our findings but will defer to the Editor’s preference if further details are desired. |
| • Address the limitations of automated translation tools for scientific terminology, by acknowledging that search engine translators may not accurately render technical language or domain-specific concepts from foreign language sources. | Thank you for this suggestion. We have added it to our limitations section (line 477-478). |
